# Flue gas desulfurization gypsum mineralization in waste Lye medium at pilot scale

**DOI:** 10.1038/s41598-025-01627-6

**Published:** 2025-05-21

**Authors:** Yuliang Cao, Tingfeng Liu, Guodong Chen, Wenyi Tan

**Affiliations:** 1 School of Mechanical Engineering, Nanjing Institute of Technology, Nanjing, 211167 Jiangsu China; 2 Xuzhou University of Technology, Xuzhou, 221018 Jiangsu China; 3 International Joint Laboratory of Green and Low Carbon Development, Jiangsu Province, China

**Keywords:** Flue gas desulfurization gypsum (FGDG), Waste Lye, Pilot scale reactor, CO_2_ mineralization, Carbon capture and storage, Solid-phase synthesis

## Abstract

CO_2_ capture, utilization and sequestration technology is currently a global research hotspot with increasing CO_2_ emission and rising atmospheric temperatures. Flue gas desulfurization gypsum (FGDG) was used to realize CO_2_ mineralization in waste NaOH lye in a pilot scale bubble tower. The effects of the ionic strength, CO_2_ flow rate, reaction temperature, and liquid level in the reactor on the properties of the mineralization products and the CO_2_ mineralization efficiency were investigated using thermogravimetric analysis, X-ray diffraction (XRD), scanning electron microscopy (SEM), and particle size analysis. The experimental results indicated that ionic strength, reaction temperature and CO_2_ flow rate significantly influenced the CO_2_ mineralization efficiency of FGDG. The CO_2_ mineralization efficiency reached 92.15% under the optimized conditions (the ionic strength: 10^−2^ mol·L^−1^, CO_2_ flow rate: 20 L·h^−1^, reaction temperature: 60 °C, liquid level: 50 cm). The liquid level has a strong effect on the particle size distribution of mineralized products. A higher liquid level promotes the formation of mineralized products with smaller particle sizes. These products consist of a single cluster of crystals and the main component is calcium carbonate. The pilot scale results demonstrate optimized evidence for CO_2_ mineralization using FGDG in waste lye. Therefore, this approach enables the comprehensive utilization of three types of waste-gas, liquid, solid- generated produced in coal-fired power plants.

## Introduction

The use of fossil energy emits large amounts of greenhouse gases (e.g., CO_2_, CH_4_, N_2_O and O_3_) due to the continuous development of global industrialization. These emissions have contributed significantly to global warming, posing a severe threat to the ecological environment as well as the human survival^1,2^. Consequently, reducing CO_2_ emissions has garnered increasing attention^3,4^. CO_2_ capture, utilization and sequestration (CCUS) is currently a necessary approach to achieve CO_2_ emission reduction^5^. Among CCUS technologies, mineral sequestration (mineralization) is particularly promising, as it generates stable carbonate products that ensure permanent, safe and efficient CO_2_ storage with minimal leakage risk^6^. This technology achieves CO_2_ mineralization by precipitating stable carbonate compounds, such as calcium carbonate and magnesium carbonate^7,8^.

Certain natural minerals and industrial solid wastes containing abundant of Ca^2+^ have been explored as feedstocks for mineralization. However, CO_2_ mineralization using natural minerals requires harsh conditions, and the associated mining, milling, and separation processes make it costly^9^. In contrast, industrial solid wastes such as fly ash^10,11^, waste gypsum^12–14^, steel slag^15^, and calcium carbide slag^16^ are considered highly promising due to their high reactivity, widespread availability, and lower costs.

Flue gas desulfurization gypsum (FGDG), a byproduct of the wet flue gas desulfurization process in coal-fired power plants, is primarily composed of CaSO_4_·2H_2_O, with a content up to 93%. Currently, China’s annual FGDG emissions are approximately 80 million tons, with a utilization rate of 78%, and an annual stockpile of around 100 million tons^17^. CO_2_ mineralization using FGDG has been extensively studied at the laboratory scale. For instance, Lee used direct wet mineralization of FGDG to investigate the effects of process parameters, such as ammonia concentration, CO_2_ flow rate, and solid-liquid ratio, on the conversion rate and CO_2_ mineralization efficiency^18^. Wing et al. proposed an ultrasonic technique to study the effects of different liquid media on the mineralization process and the crystalline morphology of the products^12,19^. Ding explored the mineralization of CO_2_ by FGDG mineralization in an NH_4_Cl-H_2_O system based on the solubility equilibrium theory between a salt solution and an insoluble electrolyte, achieving a 98% carbonation rate and demonstrating the recyclability of the carbonation reaction filtrate^20^. Wang proposed an amine-promoted carbonation process for waste gypsum, coupled with amine regeneration using bipolar membrane electrodialysis (BMED), and evaluated seven types of amines as reaction media for CO_2_ mineralization^21^. Chang et al. proposed using waste lye from steel smelting and steel slag as raw materials for mineralization to absorb CO_2_, which not only reduces water consumption but also lowers the total cost^22^. However, the waste lye utilized contains a lot of impurities, resulting in a complex composition of the product. Kumar et al. investigated carbonation of the waste lye produced by the paper industry to, achieving simultaneous CO_2_ absorption and recovery of carboxylic acids and lignin, though the CO_2_ capture performance remained unevaluated in this process^23^.

Many studies have focused on liquid alkaline media, including ammonia or organic amines, for CO_2_ mineralization, which is severely limited by the cost of liquid media. Few studies have explored the application of waste lye so far, despite its availability and cost advantages. It is well known that ionic strength positively influences reaction rates in reactors^24,25^. Altiner et al. adopted NaOH and Ca(OH)_2_ in a “two-step” carbon sequestration method, but focused primarily on performance outcomes rather than the effects of ionic strength^14^.

The study demonstrates a pilot scale system to achieve CO_2_ mineralization and to recycle product. The effects of key parameters such as ionic strength in solution, CO_2_ flow rate, reaction temperature and liquid level on the CO_2_ mineralization efficiency of FGDG were systematically investigated. And the particle size and morphology of the products was characterized by means of XRD, TG, SEM, and particle size analysis. A comprehensive utilization of three kinds of wastes (gas CO_2_, solid FGDG and liquid waste lye) emitted from the coal-fired power plants was explored Furthermore, both inherent factors (e.g., ionic strength) and external factors (e.g., reactor structure) of CO_2_ mineralization at the pilot scale are discussed in detail.

## Materials and methodology

### Materials

**Fig. 1:**
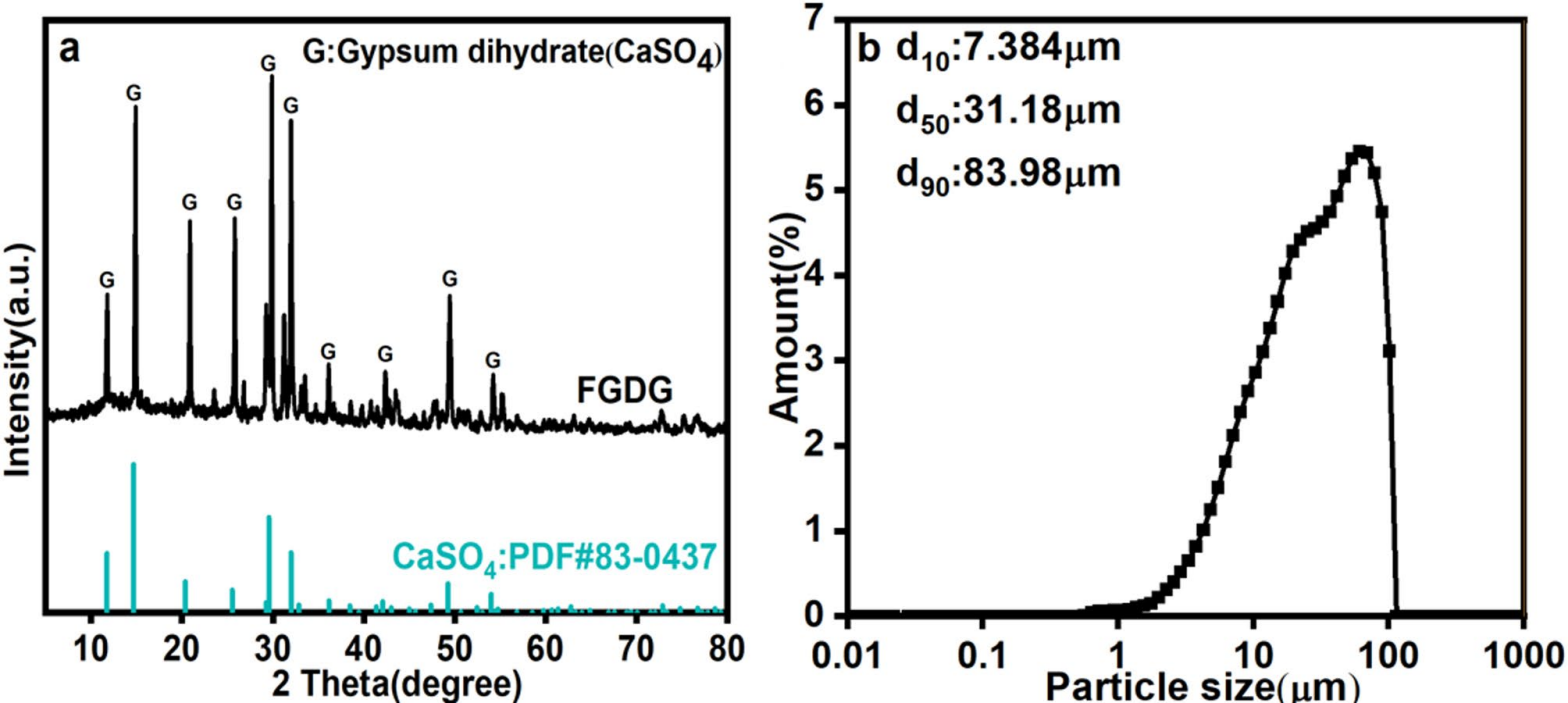
(**a**) XRD pattern of FGDG and (**b**) the particle size distribution of FGDG.

The FGDG used in this study was produced from a coal-fired power plant. The FGDG sample was first dried at 105 °C for 8 h to remove surface moisture and then sieved to 60 mesh^26^. The purity of the CaSO_4_ in the FGDG used was 97%, as determined by wet analysis. The phase composition of the samples was analyzed by X-ray diffraction (XRD, Bruker D2 Advance with a Cu Kα source at 30 kV and 10 mA) with the scanning scope (2θ) from 5° to 80° under the speed of 10° min^−1^ and the obtained patterns were examined using jade6.5 (PDF#83-0437) software for mineral identification. The XRD confirmed that the main component of the FGDG samples was calcium sulfate dihydrate, as shown in Fig. 1a. The particle size was measured with a particle size analyzer (Bettersize 3000 Plus), and the average size of the FGDG samples (d_50_) was 31.18 μm, with 90% of the particles smaller than 83.98 μm, as shown in Fig. 1b. The composition of the sample was analyzed via X-ray fluorescence (XRF, ZSX Primuss II, Rigaku), and the FGDG impurities were mainly SiO_2_ and Al_2_O_3_, as shown in Table 1. The theoretical CaO content in pure CaSO_4_·2H_2_O is ~ 32.5% (based on molar mass ratios), but the higher CaO content (46.02%) reflects the dehydration of gypsum (loss of crystalline water) during sample preparation (dried at 105 °C for 8 h) and potential partial decomposition of CaSO_4_·2H_2_O into CaO and SO_3_. This aligned with the XRD results as Fig. 1a, confirming CaSO_4_·2H_2_O as the dominant phase.

### CO_2_ mineralization experiment

NaOH solutions with different ionic strengths were prepared to simulate the waste lye, and the ionic strength of the waste lye was calculated according to the Eq. (1):

1 $$I=\frac{1}{2}\sum\limits_{{i=1}}^{n} {{C_i}{Z_i}^{2}}$$

Here, *I* is the ionic strength of the waste lye, *C*_i_ is the ion concentration, and *Z*_i_ is the charge number of ions.

**Fig. 2:**
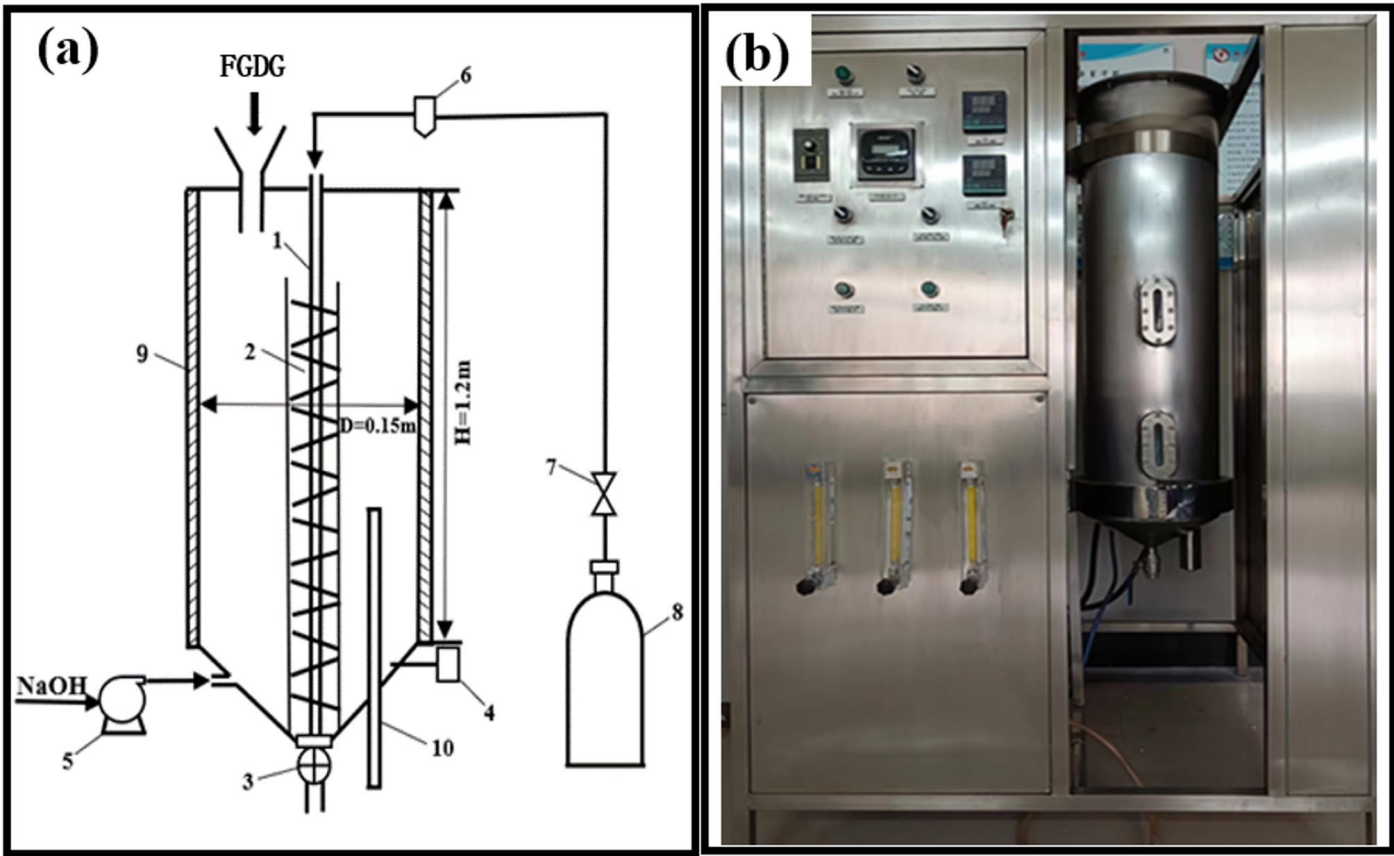
(**a**) Structure of a pilot scale mineralization reactor: 1—gas tubes, 2—heating coils, 3—valve, 4—pH meter, 5—liquid pump, 6—gas flow meter, 7—gas pressure reducing valves, 8—CO_2_ cylinders, 9—insulation, 10—sampling tube. (**b**) Physical drawings of a pilot scale mineralization reactor.

**Fig. 3:**
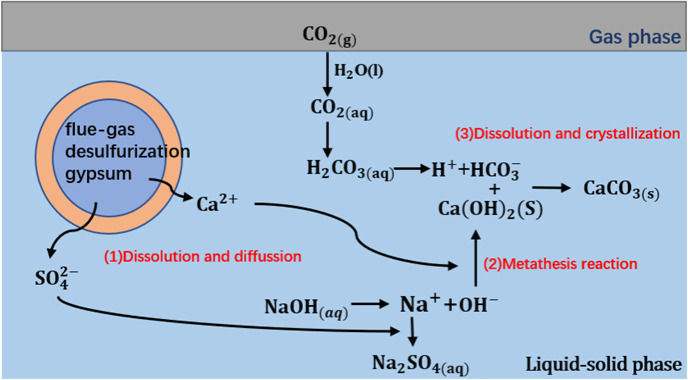
Schematic diagram of carbonization and carbon sequestration of FGDG.

The waste lye was pumped into the pilot scale reactor with a height of 120 cm, an inner diameter of 15 cm and a length of the sampling tube 50 cm, as shown in Fig. 2a. The physical diagram of the reactor is shown in Fig. 2b. After the waste lye was heated to the desired temperature, a weighed FGDG sample was added to the waste lye through the auger to prepare slurry, and then 99% CO_2_ gas was bubbled into the slurry with vigorous stirring. The mineralization reaction process is shown in Fig. 3. According to the standards for industrial production waste lye, equipment limitations and energy-saving requirements, the effects of reaction temperature (40 °C, 60 °C, 80 °C), ionic strength (10^−12^ mol·L^−1^, 5 × 10^−9^ mol·L^−1^, 10^−2^ mol·L^−1^), CO_2_ flow rate (20 L·h^−1^, 40 L·h^−1^, 60 L·h^−1^) and liquid level (30 cm, 40 cm, 50 cm) on FGDG mineralization were investigated^34^. The detailed experimental conditions are shown in Table 2. For example, for I_10_^−12^-T40-r20-h30, the experiment was carried out at I = 10^−12^ mol·L^−1^, a reaction temperature of 40 °C, a CO_2_ flow rate of 20 L·h^−1^ and a liquid level of 30 cm. Samples were taken from the exit at a given reaction time for determination (2, 4, 6, 15, 30, and 60 min), and the pH was measured at various reaction time points (2, 4, 6, 8, 10, 15, 20, 25, 30, 40, 50, and 60 min).

**Table 1:** Chemical composition of FGDG.

Material	CaO	SO_3_	SiO_2_	Al_2_O_3_	MgO	Na_2_O	K_2_O	LOI*
wt%	46.02	46.15	3.6	1.56	0.55	0.17	0.26	1.69

**Table 2:** Reaction conditions in the pilot scale experiments.

Reaction condition	1	2	3
Temperature, T/°C	40	60	80
Ionic strengths, I/mol·L^−1^	10^−12^	5 × 10^−9^	10^−2^
CO_2_ flow rate, r/L·h^−1^	20	40	60
liquid level, h/cm	30	40	50

### Characterization of the product and calculation of the storage capacity

All the samples were separated from the liquid by a high-speed centrifuge, and the obtained solid samples were dried at 180 °C for 8 h. The purity of the CaCO_3_ in the product was calculated from the weight loss of the solid samples during the thermogravimetric process which was increased from 600 °C to 780 °C under N_2_ atmosphere conditions. Pane and Lin concluded that the decomposition of CaCO_3_ occurred at 600 °C to 780°C^27,28^. A particle size analyzer (Bettersize 3000 Plus) was used to determine the particle size distribution of the mineralization products.

The morphology of the products was observed by scanning electron microscopy (National Ceremony SEM 4000). The mass of CaCO_3_ in the sample at time t is calculated via the following Eq. (2):

2 $${m_{\text{t}-\text{C}\text{a}\text{C}{\text{O}_3}}}=\frac{{\Delta {m_{\text{c}{\text{o}_2}}}}}{{{\text{M}_{\text{c}{\text{o}_2}}}}} \times {\text{M}_{\text{C}\text{a}\text{C}{\text{O}_3}}}$$

Where $\Delta {m_{{\text{c}}{{\text{o}}_{\text{2}}}}}$ is the weight of CO_2_ that escapes when CaCO_3_ decomposes during thermogravimetry and where ${m_{{\text{t}} - {\text{CaC}}{{\text{O}}_3}}}$ is the mass of CaCO_3_ in the sample of products. M_caco3_ is the relative molecular weight of CaCO_3_ and ${{\text{M}}_{{\text{C}}{{\text{O}}_2}}}$ is the relative molecular weight of CO_2_. The purity of the CaCO_3_ in the sample at time t is calculated via Eq. (3):

3 $${P_{\text{t}-\text{C}\text{a}\text{C}{\text{O}_3}}}=\frac{{{m_{\text{t}-\text{C}\text{a}\text{C}{\text{O}_3}}}}}{{{m_\text{p}}}}$$

Here, ${P_{{\text{t}} - {\text{CaC}}{{\text{O}}_3}}}$ is the purity of the CaCO_3_ in the sample of products, and *m*_p_ is the mass of the sample of products.

4 $${\rho _\text{t}}=\frac{{{P_{\text{t}-\text{C}\text{a}\text{C}{\text{O}_3}}}}}{{{P_{\text{C}-\text{C}\text{a}\text{C}{\text{O}_3}}}}}$$

Where ${\rho _\text{t}}$ represents the CO_2_ mineralization efficiency of FGDG and ${\text{P}_{\text{C}-\text{C}\text{a}\text{C}{\text{O}_3}}}$represents the purity of CaCO_3_ when FGDG was 100% mineralized. The purity of the CaSO_4_ in FGDG used in the study was 97%, as determined by wet analysis. Therefore, the purity of the reaction product should be 95.9% for 100% mineralization.

## Results and discussion

### Effect of reaction parameters on CO_2_ mineralization efficiency

#### Effect of the initial ionic strength of waste Lye on the CO_2_ mineralization efficiency

The effects of the initial ionic strength of waste lye on CO_2_ mineralization were evaluated by varying initial ionic strengths, which were 10^−12^ mol·L^−1^, 5 × 10^−9^ mol·L^−1^, and 10^−2^ mol·L^−1^ (denoted as I_10_^−12^, I_5 × 10_^−9^ and I_10_^−2^, respectively).

**Fig. 4:**
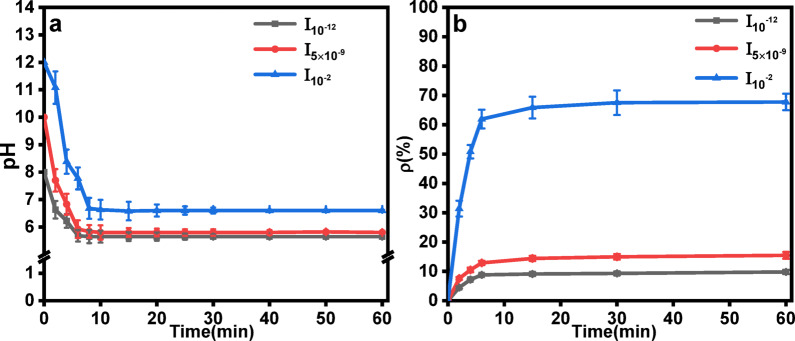
(**a**) pH value curves with respect to time and **(b**) Effect of the initial ionic strength of waste lye on the CO_2_ mineralization efficiency (Conditions: 40 °C temperature, 20 L·h^−1^ CO_2_ flow rate, 30 cm liquid level.).

Figure 4a shows the variation in slurry pH with time. As CO_2_ was introduced into the slurry, the reaction began, and OH^-^ ions in the slurry were consumed, resulting in a decrease in the pH of the slurry. The pH of the slurry gradually stabilized after approximately 6 min of continuous reaction. Under all conditions, the CO_2_ mineralization efficiency and slurry pH tended to stabilize at around 6 min, which was considered the termination point of the mineralization reaction, as shown in Fig. 4a. Higher ionic strength increases ion collision frequency and accelerates Ca^2+^ leaching from FGDG, thereby promoting mineralization kinetics at the early stage. Despite varying initial ionic strengths, the reaction is ultimately limited by CO_2_ dissolution and diffusion rates in the bubble column reactor. When the system reaches the equilibrium, the reaction process tends to be stabilized regardless of the ionic strength. At this stage, the reaction terminates regardless of initial ionic strength, as the mineralized system becomes limited by the availability of reactants rather than kinetic factors^29^.

When the ionic strength decreases, the final pH of the slurry drop due to the protonation of OH^-^ during CO_2_ dissolution:

$$\text{C}{\text{O}_2}+\text{O}{\text{H}^ - } \to \text{H}\text{C}{\text{O}_3}^{ - }\quad ({\text{pH}}\sim {\text{1}}0{-}{\text{12}})$$

$$\text{H}\text{C}{\text{O}_3}^{ - }+\text{O}{\text{H}^ - } \to \text{C}{\text{O}_3}^{{2 - }}+{\text{H}_2}\text{O}\quad ({\text{pH}\sim 7}{-}{\text{9}})$$

At higher ionic strengths (10^−2^ mol·L^−1^, the buffering capacity of the waste lye increases due to the Le Chatelier principle: excess Na⁺ shifts the carbonate equilibrium toward CO_3_^2−^ formation, maintaining a near-neutral pH (~ 7) even after OH^−^ depletion^30^. The final pH of the slurry slightly increased as the initial ionic strength of the waste lye increased. The final pH increases as the initial ionic strengths of the waste lye increases. This phenomenon is attributed to activity coefficient effects. According to the Davies equation, the activity coefficient of OH^−^ decreases at high ionic strengths due to ion pairing effects, which reduces the effective OH^−^ concentration, while the analytical (total) OH^−^ concentration remains unchanged. This phenomenon aligns with the observed pH trend^31^. The CO_2_ mineralization efficiency of FGDG was also affected by the initial ionic strength of the waste lye. After approximately 6 min of reaction, the mineralization reaction was essentially complete, and then the reaction approached the saturation state, as shown in Fig. 4b. The CO_2_ mineralization efficiency of FGDG increased slightly with the continuous influx of CO_2_ after 6 min of reaction. Increasing the ionic strength of waste lye from 10^−12^ mol·L^−1 ^to 10^−2^ mol·L^−1^ enhanced the CO_2_ mineralization efficiency from 9.8% to 67.76%. According to collision theory, when the initial ionic strength of the spent lye reaches 10^−2^ mol·L^−1^, the number of Na^+^ and OH^−^ ions per unit volume is large, leading to a higher frequency of effective collisions between different ions per unit time and an increase reaction rate^32^. Moreover, as the concentration of OH^−^ in the slurry increases, the amount of Ca^2+^ leached also increases, intensifying the mineralization reaction and resulting in higher CO_2_ mineralization efficiency Luo verified this conclusion from the perspective of pH^33^.

The above results indicated that a high ionic strength of waste lye would be beneficial for the mineralization reaction to generate higher purity CaCO_3_ in the products, and the mineralization reaction would be more thorough. A high ionic strength of waste lye is ideal for increasing CO_2_ mineralization efficiency^34,35^.

#### Effect of the CO_2_ flow rate on the CO_2_ mineralization efficiency

The effects of the CO_2_ flow rate on CO_2_ mineralization were evaluated by varying the CO_2_ flow rates which were 20, 40 and 60 L·h^−1^ (denoted as r20, r40 and r60, respectively).

**Fig. 5:**
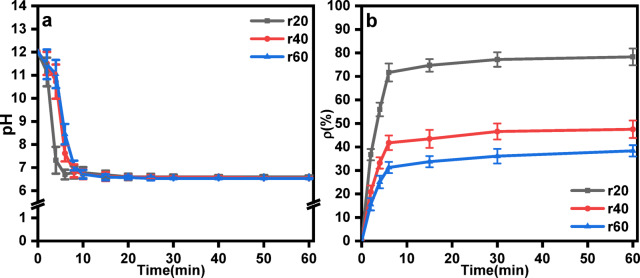
(**a**) pH value curves depending on time and (**b**) Effect of CO_2_ flow rate on CO_2_ mineralization efficiency (Conditions: 40 °C temperature, 10^−2^ mol·L^−1^ initial ionic strength of waste lye, 30 cm liquid level.).

In all the samples, the pH of the slurry decreased until it stabilized at 7 after around 10 min, as shown in Fig. 5a. The CO_2_ mineralization efficiency also tended to stabilize when the reaction lasted for about 10 min. However, the final CO_2_ mineralization efficiency decreased from 67.76% to 38.35% when the CO_2_ flow rate increased from 20 L·h^−1^ to 60 L·h^−1^, as shown in Fig. 5b. Increasing the CO_2_ flow rate was not conducive to achieving high CO_2_ mineralization efficiency of FGDG. This may be attributed to the increase in bubble diameter and the decrease in the contact area between CO_2_ and the slurry at higher CO_2_ flow rate^36,37^. According to Henry’s Law, the dissolution of CO_2_ into the liquid phase is directly proportional to the interfacial area. A reduced interfacial area limits the dissolution of CO_2_ into the waste lye, thereby decreasing the availability of dissolved CO_2_ for the mineralization reaction^38^. Additionally, the contact time between CO_2_ bubbles and the liquid phase decreased at higher flow rates. This is because the bubbles rise more rapidly through the reactor, reducing the time available for CO_2_ to dissolve and react with Ca^2+^ ions leached from FGDG, which is unfavorable for CO_2_ mineralization.

#### Effect of the reaction temperature on the CO_2_ mineralization efficiency

**Fig. 6:**
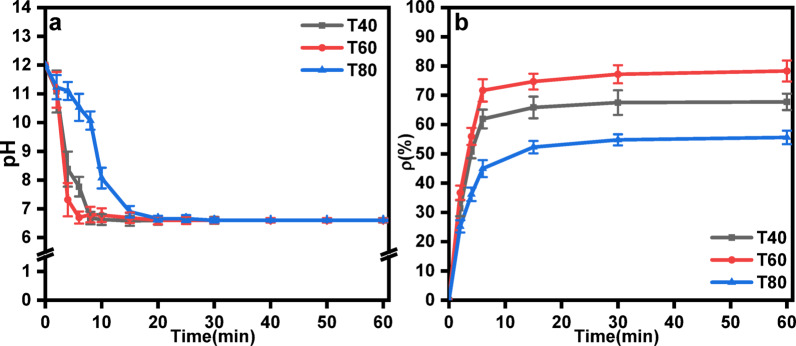
(**a**) pH value curves with respect to time and (**b**) Effect of reaction temperature on the mineralization efficiency (Conditions: 20 L·h^−1^ CO_2_ flow rate, 10^−2^ mol·L^−1^ initial ionic strength of waste lye, 30 cm liquid level.).

The effects of the reaction temperature of waste lye on CO_2_ mineralization were evaluated by varying the reaction temperature of the waste lye. The CO_2_ mineralization efficiency tended to stabilize, and the pH of the slurry reached 7 after ~ 10 min when the reaction temperature were 40 °C and 60 °C, as shown in Fig. 6a. The time which the CO_2_ mineralization efficiency and pH of the slurry reached stability increased to about 15 min at 80 °C. When the reaction temperature was increased from 40 °C to 60 °C, the CO_2_ mineralization efficiency increased from 67.76% to 78.31%. When the reaction temperature was increased from 60 °C to 80 °C, the CO_2_ mineralization efficiency decreased from 78.31% to 55.62%, as shown in Fig. 6b.

From the perspective of ion collision, when the reaction temperature increased from 40 °C to 60 °C, the collision frequency of ions increased, and the effective number of collisions per unit time increased resulting in an increase in the reaction rate^32,39^. Moreover, the increase in temperature was conducive to the precipitation of CO_2_ mineralization components in FGDG and improved the CO_2_ mineralization efficiency^36^. At the same time, the reaction rate constant (*K*) increased exponentially with temperature, as described by Eq. (5):

5 $$K=A \times {\text{e}^{\frac{{-{E_\text{a}}}}{{RT}}}}$$

where *E*_a_ is the activation energy, *A* is the pre-exponential factor, *R* is the gas constant, and *T* is the temperature. For FGDG mineralization, the activation energy barrier for Ca^2+^ release and carbonate formation was more readily overcome at elevated temperatures^40^.

However, excessively high temperatures would reduce the CO_2_ dissolution rate, and the CO_3_^2−^ concentration in the solution decreased, resulting in a decline in the reaction rate and CO_2_ mineralization efficiency. According to Henry’s Law, the solubility of CO_2_ in water decreased with rising temperature. At 80 °C, CO_2_ gas-liquid mass transfer was hindered, reducing the availability of dissolved CO_2_ for FGDG. The carbonation reaction is exothermic. Le Chatelier’s principle predicts that higher temperatures shift the equilibrium toward reactant dissociation, suppressing CaCO₃ precipitation, which is consistent with previous results in the literature^41^.

#### Effects of the liquid level on the CO_2_ mineralization efficiency

**Fig. 7:**
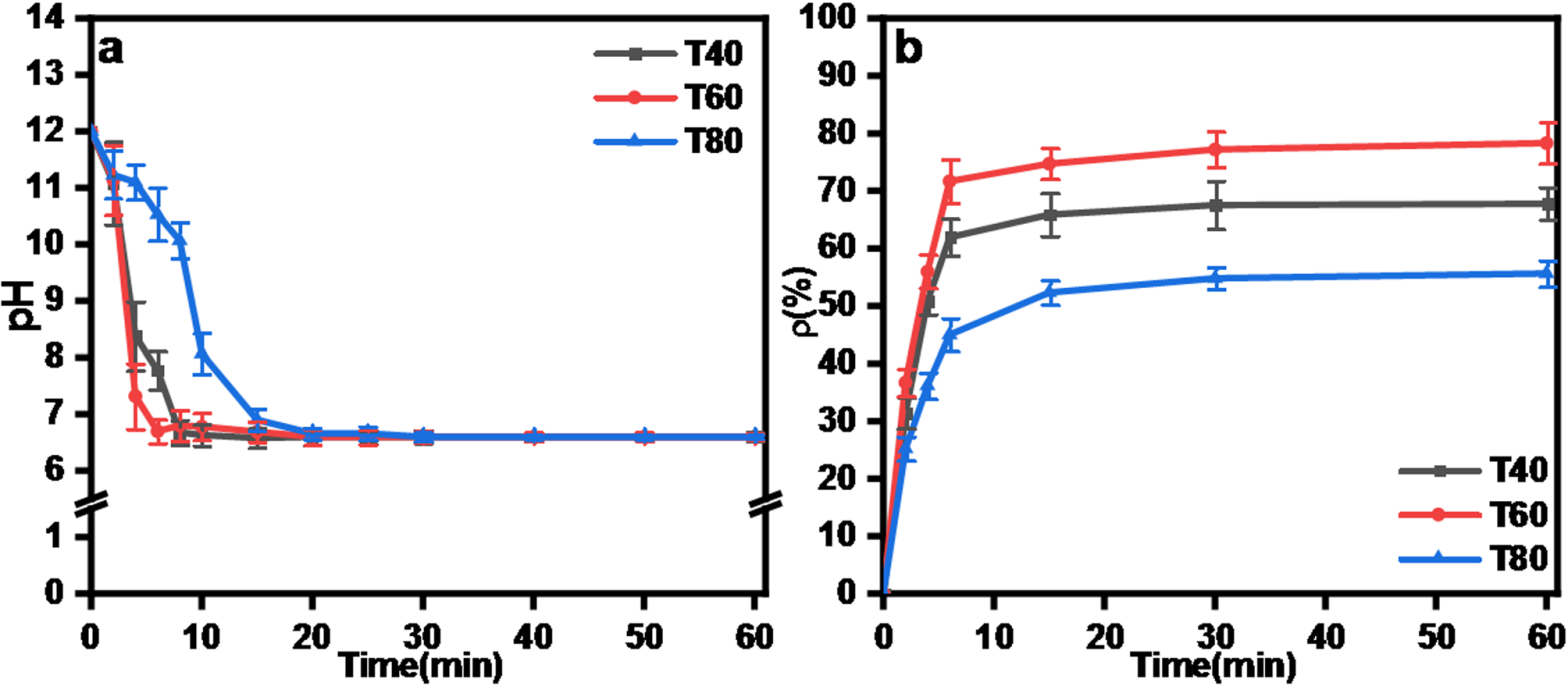
(**a**) pH value curves as a function of time and (**b**)Effect of liquid level on mineralization efficiency (Conditions: 60 °C temperature, 20 L·h^−1^ CO_2_ flow rate, 10^−2^ mol·L^−1^ initial ionic strength of waste lye.).

The effects of the liquid level of the waste lye in the reactor on CO_2_ mineralization were evaluated by varying the liquid level which was 30, 40 and 50 cm. The difference in liquid level can lead to different capacities of waste lye in the reactor during the experiment. Therefore, it was necessary to change the quantity of FGDG added, to ensure that the solid‒liquid ratio remained constant and that the experiment proceeded smoothly. The change in the liquid level was not significant. After approximately 6 min of reaction, the pH of the slurry decreased to 7, and the decline curve was consistent, as shown in Fig. 7a. At the same time, the CO_2_ mineralization efficiency tended to stabilize after about 6 min of reaction. When the liquid level increased from 30 cm to 50 cm, the CO_2_ mineralization efficiency increased from 78.31% to 92.15%, as shown in Fig. 7b. According to penetration theory, the gas-liquid contact time (*t*_c_) is proportional to the liquid level (*h*):

6 $${t_\text{c}} \propto \frac{h}{{{u_\text{g}}}}$$

where *u*_g_ is the bubble rise velocity. A taller liquid column allows CO_2_ bubbles to remain in contact with the slurry for longer, increasing the dissolution of CO_2_ and subsequent reaction with Ca^2+^ ions. This extended contact time ensures more complete conversion of CO_2_ to carbonate species (HCO_3_^−^/CO_3_^2−^), thereby improving mineralization efficiency^42^. The above results indicate that a high liquid level is favorable for high CO_2_ mineralization efficiency^43^.

### Effect of reaction parameters on product particle size

#### Effect of the initial ionic strength of waste Lye on product particle size

**Fig. 8:**
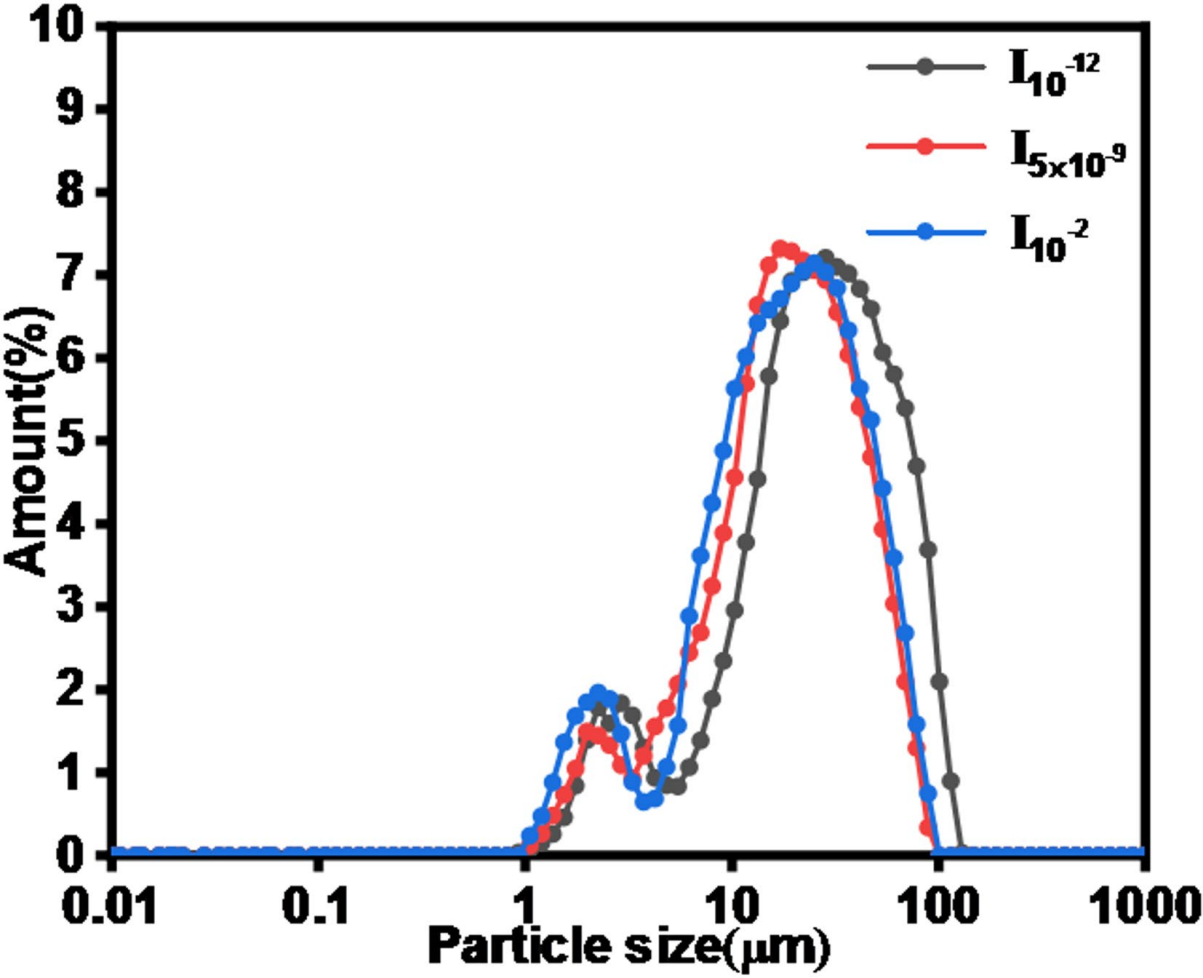
Effect of the initial ionic strength of waste lye on the particle size distribution of the products (Conditions: 40 °C temperature, 20 L·h^−1^ CO_2_ flow rate, 30 cm liquid level).

The effects of the initial ionic strength of the waste lye on the particle size distribution of the products were investigated by changing the initial ionic strength of the waste lye (10^−12^ mol·L^−1^, 5 × 10^−9^ mol·L^−1^, 10^−2^ mol·L^−1^). Figure 8 shows the particle size distributions of the products generated under different initial ionic strengths of the waste lye. The products exhibited a bimodal particle size distribution, with peak values at approximately 2 μm and 25 μm. When the initial ionic strengths of the waste lye were 10^−12^ mol·L^−1^, 5 × 10^−9^ mol·L^−1^, and 10^−2^ mol·L^−1^, the d_50_ values of the products were 13.4 μm, 11.7 μm and 10.4 μm, respectively. The d_50_ of the products decreased by 29.97% as initial ionic strength of the waste lye increased from 10^−12^ mol·L^−1^ to 10^−2^ mol·L^−1^. Higher ionic strength increased the concentration of free ions in the waste lye, accelerating the nucleation process. This resulted in a larger number of smaller CaCO_3_ nuclei forming simultaneously, which limited subsequent crystal growth due to competitive ion consumption^44^. Therefore, an increase in the initial ionic strength of waste lye is favorable for obtaining small particle products.

#### Effects of the CO_2_ flow rate on product particle size

**Fig. 9:**
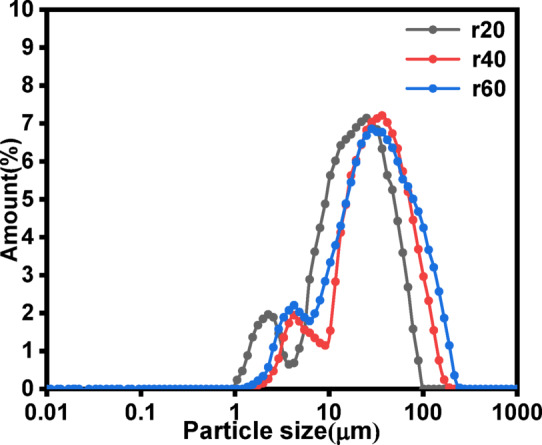
Effect of the CO_2_ flow rate on the particle size distribution (Conditions: 40 °C temperature, 10^−2^ mol·L^−1^ initial ionic strength of waste lye, 30 cm liquid level.).

The CO_2_ flow rate also significantly influenced the particle size distribution of the products. Figure 9 illustrates the effect of the CO_2_ flow rate on the particle size distribution. At a flow rate of 20 L·h^−1^, the product exhibited bimodal peaks at 1.9 μm and 28.4 μm, with a d_50_ value of 10.4 μm. When the CO_2_ flow rates were increased to 40 L·h^−1^ and 60 L·h^−1^, the bimodal peak values shifted to approximately 3.4 μm and 32.2 μm, with d_50_ values of 17.1 μm and 19.4 μm, respectively. As the CO_2_ flow rate increased from 20 L·h^−1^ to 60 L·h^−1^, the bimodal particle sizes distribution shifted to the right. And the d_50_ of the products increased by 86.55%. These results indicate that higher CO_2_ flow rates increased the proportion of large particles in the products, which hindered the formation of small particles.

A higher CO_2_ flow rate accelerated crystal growth, whereas a lower flow rate favored the formation of smaller particles. Increased CO_2_ flow rates intensified the churning and stirring of the slurry^45^, leading to greater drag forces on the particles. As a result, the larger and heavier particles were more likely to reach higher liquid levels.

Additionally, the higher CO_2_ flow rates introduced turbulence and shear stress within the reactor. According to colloidal theory, excessive turbulence can disrupt nascent CaCO_3_ crystals, resulting in a higher proportion of larger, less reactive particles. This observation aligns our findings that higher flow rates increased the particle size of the mineralization products^46^.

#### Effect of reaction temperature on product particle size

**Fig. 10:**
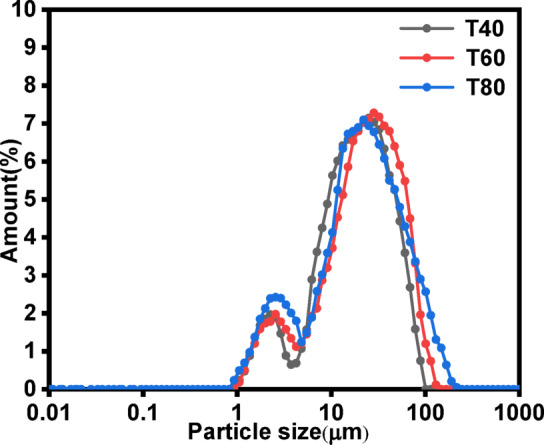
Effect of the reaction temperature on the particle size distribution (Conditions: 20 L·h^−1^ CO_2_ flow rate, 10^−2^ mol·L^−1^ initial ionic strength of waste lye, 30 cm liquid level).

Figure 10 shows the effect of the reaction temperature of waste lye on the particle size distribution of the products. Under all tested conditions, the products exhibited a bimodal distribution, with bimodal peak values ranging from 2.3 μm to 2.9 μm and 22.5 μm to 28.4 μm. The d_50_ values of the products varied between 10.4 μm and 11.3 μm. These results indicate that the reaction temperature had no significant effect on the particle size distribution of the products^47^.

#### Effect of liquid level on products particle size

**Fig. 11:**
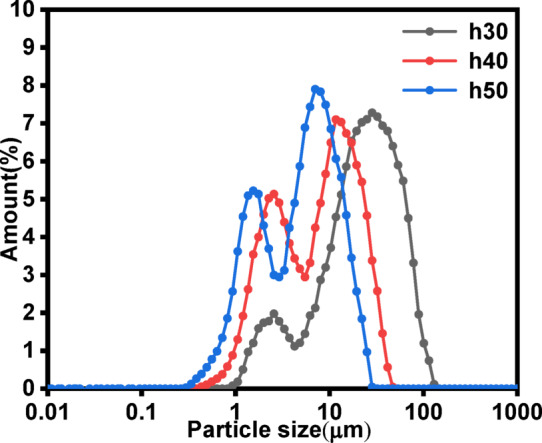
Effect of the liquid level on the particle size distribution (Conditions: 60 °C temperature, 20 L·h^−1^ CO_2_ flow rate, 10^−2^ mol·L^−1^ initial ionic strength of waste lye.).

In this stage, the influence of the liquid level on the particle size distribution of the products was studied by varying the liquid level inside the reactor. Figure 11 shows a bimodal distribution of the products under different liquid level conditions. When the liquid level was 30 cm, the bimodal peak values of the products were 1.9 μm and 28.4 μm and the d_50_ value was 10.4 μm. at a liquid level of 40 cm, the bimodal peak values shifted to 2.5 μm and 11.7 μm and the d_50_ value decreased to 6.2 μm. Further increasing the liquid level to 50 cm resulted in bimodal peak values of 1.5 μm and 7.1 μm, with a d_50_ value of 4.2 μm. When the liquid level was raised from 30 cm to 50 cm, a 62.83% reduction in the d₅₀ of the products was observed. Additionally, the peaks corresponding to smaller particle sizes became more pronounced, and the overall particle size distribution shifted to the left as the liquid level increased. The liquid level affects the turbulent shear stress (*τ*) within the reactor, which plays critical role in crystal nucleation and growth^48^.

7 $$\uptau \propto \uprho \text{u}{\prime ^{\text{2}}}$$

Where ρ is fluid density and *u*′ is turbulent velocity fluctuation. At higher liquid levels, increased turbulence generates stronger shear forces. These enhanced shear forces disrupt the growth of CaCO_3_ crystals, promoting nucleation over Ostwald ripening. This results in smaller particle sizes, as observed in our experiments. Furthermore, higher liquid levels reduce the settling velocity of FGDG particles (as described by Stokes’ law), maintaining a homogeneous slurry. This prevents particle agglomeration and maximizes the reactive surface area of CaSO_4_·2H_2_O for Ca^2+^ leaching^49^. The above experimental results demonstrate that a higher liquid level reduces the proportion of large particles in the products, leading to smaller particle sizes.

### Characterization

#### Characterization and analysis of the product

**Fig. 12:**
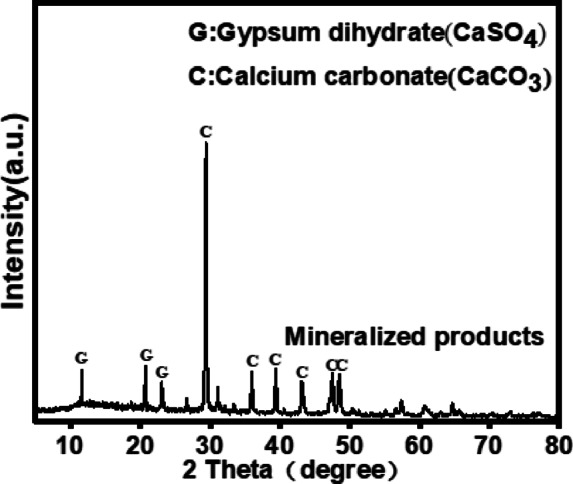
XRD patterns of the mineralized product.

**Fig. 13:**
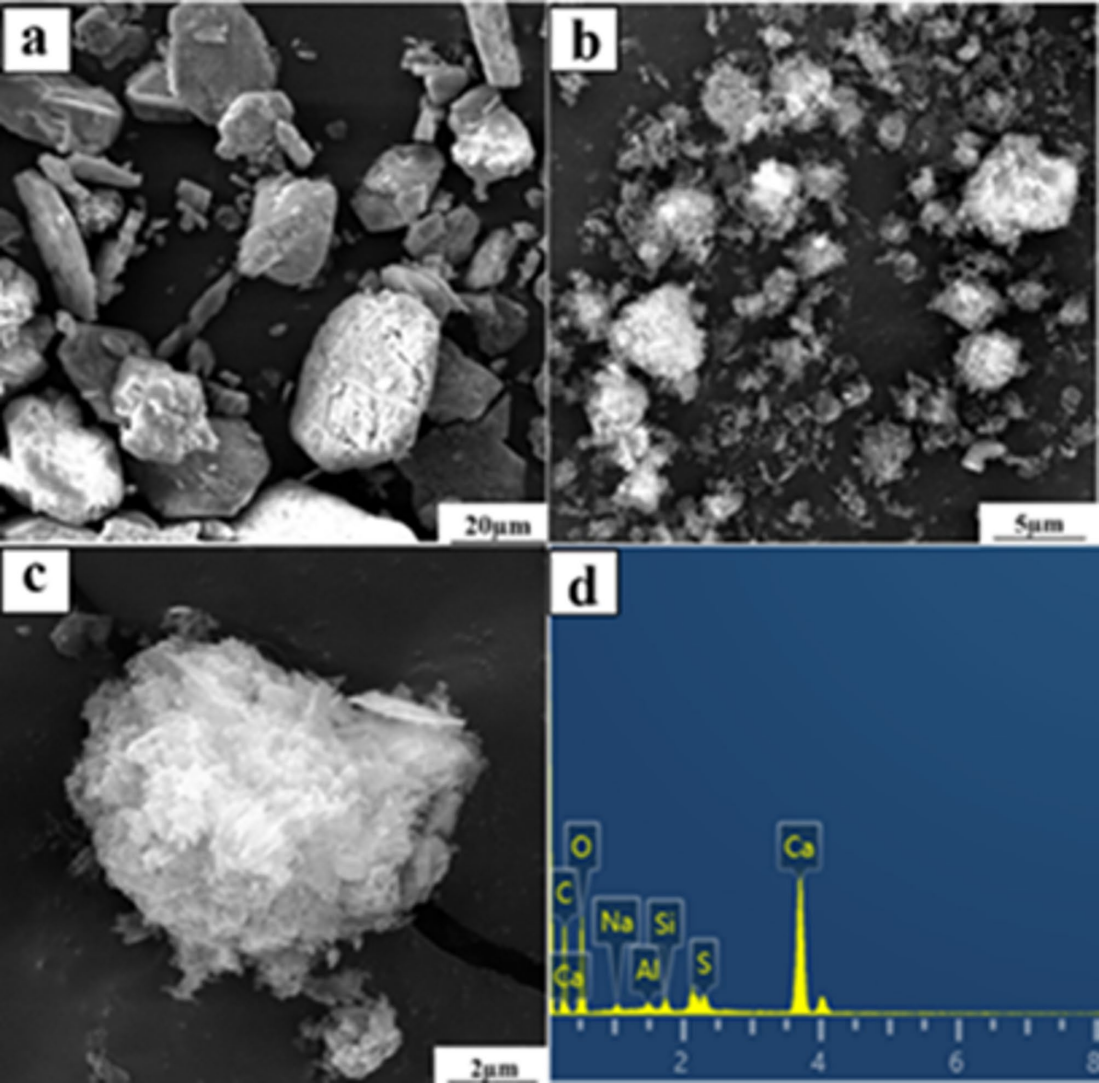
(**a**) SEM images of raw FGDG; (**b**,**c**) SEM images of mineralization products; (**d**) EDS mapping of mineralization products.

After 60 min of mineralization, CaSO_4_, the main component of dried FGDG, was converted to CaCO_3_, the main component of the mineralization product. However, a small amount of CaSO_4_ remained in the mineralization product, as shown in Fig. 12. In gas-liquid reactor systems, gypsum particles undergo Stokes sedimentation, leading to a significant increase in particle concentration within the bottom region and forming a diffusion-limited reaction zone. Turbulent characteristics within the reactor (e.g., Taylor-scale vortices) influence particle collision frequency and mass transfer efficiency, with this process exhibiting multi-scale coupling effects. Therefore, the variation of the proportion of incomplete CaSO_4_ conversion and its potential impact on product properties are complex and multifaceted^50^. Figure 13a presents a scanning electron microscope (SEM) image of the FGDG. The carbonization products consisted of amorphous nanosized particles with an aggregate structure^18,51^, in contrast to the original diamond-shaped morphology of FGDG, as shown in Fig. 13b, c. Energy-dispersive X-ray spectroscopy (EDS) analysis of the carbonized products, shown in Fig. 13d, revealed that they primarily contained Ca, C, and O, confirming the formation of CaCO_3_ aggregates.

## Conclusion

The study was focused on that FGDG mixed with waste NaOH lye can function as an absorbent to capture CO_2_ and then CaCO_3_ subsequently produced through the gas-liquid- solid phase reaction. The CO_2_ mineralization efficiency increased from 9.8% to 67.76% and the d_50_ of the products decreased from 13.4 μm to 10.4 μm with the elevated ionic strength. Reducing the CO_2_ flow rate can prolong the gas-liquid-solid contact time, improved the CO_2_ mineralization efficiency from 38.35% to 67.76%, and reduced the d_50_ of the products from 19.4 μm to 10.4 μm. The reaction temperature significantly influenced the CO_2_ mineralization process. The CO_2_ mineralization efficiency initially increased and then decreased with rising reaction temperature, reaching an optimal efficiency of 78.31% at 60 °C. However, the reaction temperature had no significant effect on the particle size of the products. The elongated sampling tube can increase the liquid level, increased the CO_2_ mineralization efficiency from 78.31% to 92.15%, and reduced the d_50_ of the products from 10.4 μm to 4.2 μm. The maximum CO_2_ mineralization efficiency was 92.15% and the d_50_ of the products was 4.2 μm under optimized conditions (ionic strength: 10^−2^ mol·L^−1^, CO_2_ flow rate: 20 L·h^−1^, temperature: 60 °C, liquid level: 50 cm). The solid in the slurry was also transformed from columnar CaSO_4_ crystals into clusters of CaCO_3_ aggregates. In conclusion, this study successfully demonstrated an efficient method for CO_2_ mineralization using FGDG and waste NaOH lye, offering a sustainable solution for carbon capture and utilization.

## Data Availability

All data generated or analysed during this study are included in this published article.
